# Hepatitis B Vaccination: A Historical Overview with a Focus on the Italian Achievements

**DOI:** 10.3390/v14071515

**Published:** 2022-07-11

**Authors:** Luisa Romano’, Alessandro R. Zanetti

**Affiliations:** Dipartimento di Scienze Biomediche per la Salute, Università degli Studi, 20133 Milano, Italy; alessandro.zanetti@unimi.it

**Keywords:** hepatitis B, HBV, vaccine, vaccination, immunological memory, humoral immunity

## Abstract

Vaccination is the most effective way to control and prevent acute and chronic hepatitis B, including cirrhosis and HCC, on a global scale. According to WHO recommendations, 190 countries in the world have introduced hepatitis B vaccination into their national childhood immunization programs with an excellent profile of safety, immunogenicity, and effectiveness. Following vaccination, seroprotection rates are close to 100% in healthy children and over 95% in healthy adults. Persistence of anti-HBs is related to the antibody peak achieved after vaccination. The peak is higher the longer the antibody duration is. Loss of anti-HBs does not necessarily mean loss of immunity since most vaccinated individuals retain immune memory for HBsAg and rapidly develop strong anamnestic responses when boosted. Evidence indicates that the duration of protection can persist for at least 35 years after priming. Hence, booster doses of vaccines are currently not recommended to sustain long-term immunity in healthy vaccinated individuals. In Italy, vaccination against hepatitis B is met with success. In 2020, Italy became one of the first countries in Europe to be validated for achieving the WHO regional hepatitis B control targets.

## 1. Introduction

Hepatitis B is still a major worldwide public health problem. Globally, 296 million people are estimated to be chronic carriers of hepatitis B virus (HBV), with approximately 820,000 deaths per year for complications mainly due to HBV-related cirrhosis and hepatocellular carcinoma (HCC) [1]. According to the 2016 WHO call, all countries are invited to work together in the effort to eliminate viral hepatitis B and C as a public health threat by the year 2030 [2]. To achieve this goal, vaccination is one of the most effective measures and a cost-effective option to control and prevent hepatitis B and its long-term sequelae on a global scale. The success of a program of vaccination depends on the availability of safe and highly effective vaccines and on the implementation of proper strategies of vaccination.

## 2. Hepatitis B Vaccines

The first vaccines against hepatitis B, known as plasma-derived vaccines, were developed in France and in the USA in the early 1980s by harvesting the 22 nm hepatitis B surface antigen (HBsAg) particles taken from the blood of carriers of HBsAg, which were then inactivated and purified through treatments with a combination of urea, pepsin, formaldehyde, and heat. These first-generation vaccines have been administered to several million individuals with an excellent record of safety and effectiveness [3,4] (Table 1).

**Figure 1:**
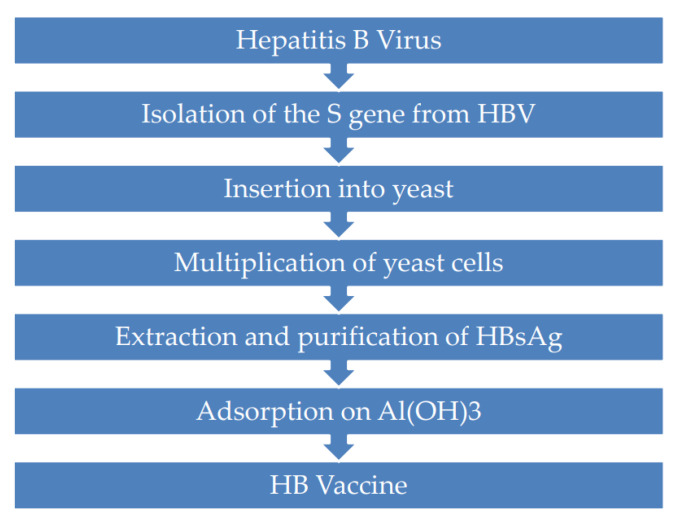
Key steps for preparation of hepatitis B recombinant vaccine.

Despite this success, concern grew both in Europe and North America about the belief that the plasma used for vaccine preparation could be contaminated with other blood-borne viruses such as HIV (human immunodeficiency virus), which shares several modes of transmission with HBV. Though no cases of HIV or any other infection due to HB (Hepatitis B) vaccination have been documented, unfounded fears expressed about the safety of plasma-derived vaccines limited the use of such vaccines that were largely replaced around the mid-1980s by recombinant DNA vaccines (rec-DNA vaccines). Vaccines developed with this new technology (second-generation vaccines) were manufactured by the expression of HBsAg protein in genetically engineered mammalian cells or in yeast cells (*Saccharomyces cerevisiae*) containing the HBV Surface gene (S gene) [5,6]. In particular, preparation of the yeast-derived vaccine involved a series of multiple steps which included the isolation of the S gene from the HBV and its insertion into yeast cells, which multiply during a process of fermentation to produce large amounts of HBsAg. After extraction and purification, the expressed HBsAg polypeptides self-assemble into spherical non-infectious particles almost identical to the 22 nm particles found in the serum of HBsAg carriers. The next step is the adsorption of the HBsAg on aluminum hydroxide or Al(OH)_3_, which acts as an adjuvant to enhance the vaccine immunogenicity. This highly sophisticated new genetically engineering technology offered the potential to produce unlimited supplies of vaccines, allowing the extension of vaccination on a global scale (Figure 1).

During the 1990s, third-generation HB vaccines were produced in HBV-transfected mammalian cells, such as CHO (Chinese hamster ovary) or mouse-derived cell lines which express and secrete the small S and the middle pre-S2 glycoproteins or all three viral envelope proteins (i.e., S, pre-S1, pre-S2). Evidence indicates that vaccines containing pre-S/S proteins can induce a higher and faster antibody response compared to yeast-derived S-containing vaccines, and thus, they are potentially more effective in vaccinating people who are non-responders or poor responders to conventional vaccines [7,8,9]. Recently, two phase 3, double-blinded, randomized, multicenter studies demonstrated that a 3-antigenic hepatitis B vaccine (TAV, containing S, pre-S1, and pre-S2 antigens) was able to elicit a robust immune response noninferior in healthy adults aged 18 years or older and was superior in individuals aged 45 years or older compared to a mono-antigenic vaccine (MAV, containing S antigen) [10,11]. In addition, the rapid and high seroprotection rates achieved after the administration of TAV suggest that this vaccine can offer seroprotection to more adults earlier than MAV. These findings could be of clinical relevance, especially for the substantial number of adult non-responders to conventional hepatitis B vaccines, those at higher risk of hepatitis B infection, and those more prone to develop severe complications associated with HBV infection.

To make easier the introduction of HB vaccination into the existing programs of immunization targeted to infants and children, a wide array of formulations of combination vaccines were then developed. Among these, the hexavalent vaccines, or the six-in-one vaccine, able to simultaneously protect against six diseases (diphtheria, tetanus, pertussis, poliomyelitis, hepatitis B, and invasive infections caused by *Haemophilus influenza type b*) were authorized in the European Union in October 2000 for primary immunization of infants in the first year of life (three doses administered at 1–2 months intervals from 2 months of age) and as a booster dose given 6–12 months after the end of primary vaccination series. It has been shown that for each of these combinations, the various antigenic components remain highly immunogenic, safe, and well tolerated, facilitating the vaccination schedules through the administration of broadly effective vaccines in a reduced number of injections. This means less infant discomfort with the additional advantages of favoring parent compliance and improving the achievement and maintenance of adequate vaccination coverage rates at lower costs.

Combination vaccines containing hepatitis A and hepatitis B antigens have been prepared with success to provide dual protection against both diseases. Persons at increased risk of acquiring hepatitis A and B, such as travelers from low to high endemic areas and military personnel, take particular benefit from the introduction of such vaccines, whose immunogenicity and safety were shown to be equal to the separate administration of hepatitis A and B monovalent vaccines [12,13].

Novel adjuvants have been prepared with the aim of improving the immune response against HBsAg in special groups of non-responders or hypo-responders to conventional hepatitis B vaccines. Among them, a vaccine with enhanced immunogenicity, containing HBsAg adjuvated with aluminum phosphate and 3-O-desacyl 1-4′monophosphoril lipid A (MPL), has been successfully developed and used in several countries for active immunization of patients with an impaired immune system, such as those with renal insufficiency, including pre-hemodialyzed and hemodialyzed patients, and liver transplanted patients [14,15]. Recently, the FDA and EMA approved a recombinant monovalent HBV vaccine adjuvated with CpG (cytosine phosphoguanosine), able to stimulate innate immunity through TLR9 [16]. This new vaccine, currently approved only for adults, requires two doses over one month. The characteristic of this new vaccine (i.e., shorter schedule, earlier seroprotection, higher adherence and seroprotection rate) makes it an additional option for HB vaccination of hypo/non-responders individuals.

## 3. Vaccination

With the advent of hepatitis B vaccines, strategies of vaccination in industrialized countries were initially targeted at the immunization of people at higher behavioral or professional risk of exposure to HBV infection, including healthcare workers, men who have sex with men (MSM), people with multiple sexual partners, intravenous drug users (IVDUs), household contacts of chronically infected persons, babies born to HBsAg carrier mothers, and to some defined patient subgroups as hemophiliacs and polytransfused patients, and those on hemodialysis maintenance. Vaccination of such high-risk groups had an individual benefit but a little or no impact in terms of control and prevention of hepatitis B in the population at large. Thus, the failure of such policies of vaccination in lowering the burden of hepatitis B led the WHO’s Global Advisory Group of the Expanded Program of Immunization to recommend to all countries the integration of universal infant or adolescent (or both if economically feasible) hepatitis B vaccination into their national immunization programs by 1997 [17]. At present, 190 countries worldwide have implemented this recommendation.

### 3.1. The Italian Strategy

**Figure 2:**
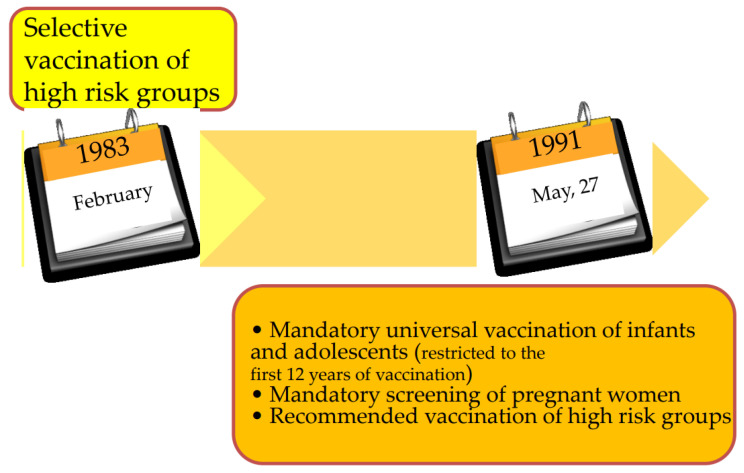
The Italian strategies for hepatitis B vaccination.

In Italy, vaccination started in 1983 and was first addressed to people at higher risk of HBV infection, particularly healthcare workers, IVDUs, babies born to HBsAg carrier mothers, and hemodialyzed patients. In 1991, universal vaccination became law for all infants and for all 12-year-olds, with the latter restricted to the first 12 years of vaccination [18]. Thus, at the end of 2003 (or 12 years after the implementation of this program of immunization), vaccination of adolescents was stopped (since children at this age were already covered), while that of infants was maintained (Figure 2).

Mandatory screening for HBsAg of pregnant women to identify babies needing to be simultaneously treated with HBIg (hepatitis B immunoglobulin) and vaccine at birth, and recommendations for vaccination of people whose occupation or lifestyle exposed them to a higher risk of HBV infection were additional key points of this program.

In Italy, hepatitis B vaccination was well received because of high public awareness of the disease and its consequences. The uptake of vaccine was rapid and reached in a short time a coverage rate of around 95%, with some yearly fluctuations and geographic differences, with higher rates in the northern regions compared to the southern regions of the country [19]. As a result of this policy, over 25 million Italians 42 years of age or younger (i.e., 42 birth cohorts) had been immunized by the end of 2021 with success against hepatitis B.

### 3.2. Immunogenicity and Safety

Following a full course of vaccination with the classical schedule of three doses of vaccine given at 0, 1, and 6 months, seroprotection rates of anti-HBs at concentration equal to or higher than 10 mIU/mL (the antibody threshold considered protective) are close to 100% in healthy children and almost 95% in healthy adults, but less in patients with an impaired immune system (including those infected with HIV) and people who are elderly, obese, and heavy smokers [20,21,22,23,24]. Patients undergoing hemodialysis or immunosuppressant therapy require higher doses of the vaccine and more injections to achieve protection [14,15]. The administration of adjuvated vaccines and the administration of recombinant triple antigen (pre-S1, pre-S2, and S) vaccines seem to be effective in enhancing the immune response in such patients [9].

Along with factors related to the host (i.e., age, gender, immunocompetence, genetics, co-infections), factors related to the vaccine and vaccination have also been found to affect the response to vaccination. Among these, dosage and schedule of vaccination, site of injection, and route of administration are key factors for achieving an optimal immune response. The amount of HBsAg protein per dose that induces a protective immune response varies by manufacturer and is partially due to the production processes of purification and inactivation used to formulate the various vaccines. In general, the vaccine dosage for infants is 50% lower than that required for adults.

Two primary immunization schedules are usually recommended to reach optimal protection: a rapid schedule with immunization at 0, 1, and 2 months, which confers protection more quickly, and schedules with more distance between the second and third dose, such as immunization at 0, 1, and 6 months, which takes longer to induce protection but produces higher anti-HBs antibody titers. Hepatitis B vaccine is given intramuscularly into the deltoid region for children and adults and into the anterolateral thigh muscle for neonates. Antibody response is lower when the vaccine is injected in the buttock [20]. Subcutaneous administration is only indicated in patients with severe bleeding disorders (i.e., hemophiliacs), and intradermal administration is not recommended [25].

Several follow-up studies showed that hepatitis B vaccination has an outstanding safety profile. Side effects (erythema, induration, and swelling) are generally mild, transient, and confined to the site of injection. Systemic reactions including fatigue, low-grade fever, headache, malaise, rash, urticaria, abdominal pain, arthralgia, and myalgia are uncommon.

In 1998, case reports from France raised concern in the French public that hepatitis B vaccination may be linked to new cases of flare-ups of multiple sclerosis (MS) or other demyelinating diseases such as Guillain Barré syndrome. This concern has led to significant reductions in the uptake of hepatitis B vaccine in France. Following the cautionary principle, the Ministry of Health temporarily suspended the hepatitis B school-based immunization program for pre-adolescents in October 1998. However, based on the analysis of the French pharmacovigilance data and the extensive review of the results derived from specific epidemiological studies, no causal relationship between hepatitis B vaccination and MS was established [26,27,28]. In 2004 and in 2009, two case control studies carried out by Hernan et al. and by Mykaeloff et al. came back to the link between vaccination and increased risk of developing MS [29,30]. However, this association was further rebutted by the WHO’s Global Advisory Committee on Vaccine Safety, which stated that evidence derived from these studies was insufficient to support such a cause–effect link, and thus not convincing enough to justify discontinuation of the established immunization program of vaccination, the benefit of which was universally well demonstrated [31].

Vaccination is not contraindicated in pregnant or lactating women. The only absolute contraindication is in subjects with known hypersensitivity to any component of the vaccine, or subjects having shown signs of hypersensitivity after a previous administration.

### 3.3. Long-Term Protection and Need for Booster

The success of universal infant vaccination relies on the fact that the vaccine-induced immunity can last during adolescence and adulthood, when the risk of exposure to HBV increases significantly. The duration of immunity conferred by a complete course of primary vaccination is still an issue under debate that may have wide implications when setting national vaccination policy. Questions in need of a definitive answer include: how long is post-vaccination immunity expected to last? Is the immunity depending on the persistence of anti-HBs antibodies at protective concentrations (≥10 mIU/mL)? Is booster vaccination necessary to sustain immunity?

In immunocompetent vaccinees, an anti-HBs concentration of 10 mIU/mL or more, measured ideally 2–3 months after the administration of the last dose of the primary vaccination course, is considered a reliable marker for primary immune response against the onset of acute symptomatic disease and chronic hepatitis B infection [32]. Evidence indicates that the anti-HBs concentration achieved after primary immunization is a strong predictor of antibody duration and long-term protection [33,34,35]. The persistence of anti-HBs antibodies correlates with the peak level immediately developed after primary immunization. In other words, the higher the peak is, the longer the antibodies will persist. Several follow-up studies show that vaccine-induced antibody levels decline over time, rapidly within the first year of vaccination and more slowly thereafter. The decrease follows similar kinetics regardless of the magnitude of the peak antibody level [36,37]. However, although the antibody tends to wane over time, its loss does not necessarily mean loss of protection since the immunological memory to HBsAg can outlast the antibody detection [33,34,38,39]. Indeed, vaccinated individuals who lost seroprotective levels of anti-HBs antibodies still maintain T-cell memory able to trigger anti-HBs production by B-cells when activated by revaccination or by natural exposure to HBV. Thus, vaccinees who lost antibodies usually develop rapid and strong anamnestic responses when boosted (the so-called boostability) or naturally exposed to HBV. Thus, there is no evidence suggesting that healthy vaccinees lose immunity simply because the level of anti-HBs antibodies falls below 10 mIU/mL. In other words, a negative anti-HBs result does not automatically means loss of protection, since most vaccinees retain long-term immune memory and would rapidly develop a strong anamnestic response when boosted even decades after primary vaccination [38,40,41,42,43,44,45,46,47,48,49].

In long-term follow-up studies, benign breakthrough infections usually characterized by anti-HBc seroconversion in the absence of HBsAg positivity are occasionally reported, but cases that develop clinical hepatitis B or chronic carrier state are very rare and generally confined to those not vaccinated properly. A recent follow-up study carried out by Bruce et al. in Alaska showed evidence of protection (presence of anti-HBs ≥10 mIU/mL or rapid response to a booster dose) in 86% of vaccinees with no clinically acute disease or development of chronic HBV infections, 35 years after priming [50]. This finding is similar to those derived from other long-term studies carried out in endemic countries such as China and The Gambia [51,52].

Indeed, in immunocompetent individuals, the longevity of specific immunologic memory to HBsAg can persist beyond the loss of humoral immunity conferring effective long-term protection. Thus, except for some higher-risk groups (e.g., healthcare workers exposed to HBV and immunocompromised patients), periodic post-vaccination testing and booster administration of vaccine when the anti-HBs antibody level falls below 10 mIU/mL are not currently regarded as necessary in routine immunization programs [38,39,40,41,43,53,54]. Whether a primary course of vaccination without the administration of booster(s) can confer life-long immunity is not known.

## 4. The Issue of Hexavac Vaccine

In Italy, starting from 1999, monovalent vaccines were gradually replaced by combined vaccines, including two hexavalent vaccines (Hexavac, Sanofi Pasteur MSD and Infanrix Hexa, Glaxo Smith Kline) introduced at the end of 2000. Such vaccines are able to simultaneously provide protection against hepatitis B as well as diphtheria, tetanus, pertussis, poliomyelitis, and invasive infections caused by *Haemophilus influenza type b.* In September 2005, in compliance with the European Medicines Agency (EMA) recommendations, the AIFA (Italian Medicines Agency) ordered, for precautionary reasons, the provisional suspension of Hexavac, as concerns were raised about its ability to provide long-term protection against hepatitis B; the license of this vaccine was definitively withdrawn in 2012 [55]. No actions were undertaken over Infanrix Hexa since immunogenicity of its hepatitis B component did not raise equal concern. Until its suspension, about 10 million doses of Hexavac were distributed globally, of which almost 90% were used for immunization of infants in Germany, Austria, and Italy. Studies carried out with Hexavac showed that although 95% of children immunized with the vaccine had protective antibody levels when tested one month after the primary course of vaccination, the antibody concentration was low (between 10 and 100 mIU/mL) in at least 5–20% of them [56]. The results of two studies conducted by Sanofi Pasteur MSD showed a lower than expected response to a booster dose of the monovalent vaccine HBVaxPRO in children who were given a primary course of Hexavac or Primavax (DT-HB vaccine), as they did not retain seroprotective antibody levels 7–9 years after primary vaccination. In addition, lower than expected anti-HBs seroconversion rates and antibody geometric mean concentrations (GMCs) were observed in children who were concomitantly given Hexavac and a seven-valent pneumococcal conjugate vaccine (Prevenar, Wyeth) or Hexavac and a meningococcal type C conjugated vaccine (NeisVac-C, Baxter) [55,57]. Thus, because of the correlation between the duration of protection and peak of the anti-HBs antibody level achieved after primary immunization, this finding raised concern that the low post-primary antibody concentrations elicited in children vaccinated with Hexavac could not provide sufficient protection against HBV during adolescence and later in life, possibly due to the loss of the immunological memory. This point deserves much consideration in Italy, where Hexavac was given to several cohorts of babies in the 5-year interval between its introduction and withdrawal. Studies carried out in Italy 3, 5, and 10 years after priming with Hexavac showed that most vaccinees who lost protective levels (<10 mIU/mL) of anti-HBs over time developed a strong anamnestic response when boosted with a single dose of monovalent hepatitis B vaccine [58,59,60,61]. In particular, an Italian open label, controlled, multi-center trial was conducted with 751 healthy pre-adolescents (aged 11–13 years) who were given either Hexavac (*n* = 409) or Infanrix Hexa (*n* = 342) at 3, 5, and 11 months of life [61]. A challenge dose of a monovalent hepatitis B vaccine (HBVaxPRO, 5 µg) was given to all participants. One month post-challenge, 83.6% of vaccinees in the Hexavac cohort and 96.4% of those in the Infanrix Hexa had anti-HBs concentrations >10 mIU/mL. Before the challenge dose, protective levels of antibodies were found in 23.9% (Hexavac cohort) and in 69% (Infanrix Hexa). In the subset of children with a pre-challenge anti-HBs concentration below 10 mIU/mL, 78.7% in the Hexavac cohort and 88.7% in the Infanrix Hexa cohort achieved post-challenge protective anti-HBs. Pre- and post-challenge antibody GMCs were higher among those vaccinated with Infanrix Hexa compared with those vaccinated with Hexavac. Data from this study suggest that Hexavac, although less immunogenic than Infanrix Hexa, induced immune memory in a high percentage of children at least 10 years after primary vaccination. Therefore, we can infer that if a vaccinated child is exposed to HBV, the immune memory rapidly induces a strong amnestic response which prevents acute disease and the development of a chronic carrier state. Thus, routine booster doses of hepatitis B vaccine were not deemed necessary to sustain immunity in children vaccinated with Hexavac. This conclusion was specific to the check performed 10 years after priming. However, continued surveillance is required to monitor whether immunological memory in such vaccinated children persists over time during teen years and adulthood when the risk of HBV exposure does significantly increase or whether a booster might be needed later in life to confer long-lasting protection.

## 5. Effectiveness of Vaccination

Vaccination has clearly proven to be effective in reducing the incidence of the disease, carrier rates, and HBV-related mortality. Taiwan is one of the best examples of a previously highly endemic country which benefited following mass vaccination, launched in 1984. In Taipei, the HBsAg prevalence in children decreased from 9.8% in 1984 to 0.4% in 2019 [62,63]. Moreover, the annual average incidence of HCC declined significantly in vaccinated children and teenagers compared to those not vaccinated [64,65,66]. Similar successful results were achieved with the introduction of vaccination in other hyper-endemic places like Alaska and The Gambia [67,68].

In the past three decades, the burden of hepatitis B has dramatically declined in Italy as a result of social, behavioral, and demographic changes; the general improvement in standard of living and hygiene; and the introduction of effective public health measures including educational campaigns against HIV infection, refinement in blood screening, use of universal precautions in medical settings, and, of utmost importance, the implementation of vaccination policies.

In Italy, in the late 1970s to early 1980s, hepatitis B was a major cause of morbidity and mortality, with at least 7500 yearly newly acquired acute hepatitis B, an estimated 1.5–2 million chronic carriers of HBsAg (i.e., about 3% of the inhabitants), and approximately 9000 deaths per year from HBV-related disease such as cirrhosis and liver cancer [69]. Because of under-reporting, and because most HBV infections are asymptomatic and go unnoticed to the surveillance system, we estimate that the actual number of people infected with HBV yearly was, at that time, at least five to ten times higher than that reported. The number of cases was higher in males than in females, in North/Central Italy than in the South Islands, and the highest attack rate was among 15–24-year-olds, reflecting their increased behavioral risks (i.e., parenteral drug use, having unprotected sexual activity with multiple sexual partners) of exposure to HBV.

The introduction of vaccination in the early 1980s, first targeting people at higher risk of infection and then all infants and 12-year-olds, has significantly changed the face of HBV epidemiology in Italy. According to SEIEVA, the overall incidence rate of acute viral hepatitis B has fallen in the past 30 years from 5 per 100,000 people in 1990 (i.e., the year before the implementation of universal vaccination) to 0.18 per 100,000 people in 2021, a 96.4% reduction [70]. The fall in this interval of time was even more striking in people aged 15–24 years in whom the morbidity rate per 100,000 inhabitants dropped from 17 to 0.02 (99.9% reduction) and from 1 per 100,000 people to zero cases (100% reduction) in those aged 0–14 years. As recently reported, the vast majority (96.8%) of the over 11,000 acute viral hepatitis B cases notified to SEIEVA over 22 years of surveillance (1993–2014) were unvaccinated; only a very small proportion (0.4%) of those who were successfully vaccinated acquired the clinical disease, indicating that the vaccination failure is a rather rare event [71]. Molecular analysis of HBV derived from 13 patients who were fully vaccinated showed that seven of them were infected with the wild-type virus and six by S-gene mutants, including the prototype mutant virus with glycine to arginine substitution (G145R) [72]. About one-third of notified cases of acute HB were not vaccinated, despite being well aware to be at behavioral or at occupational risk of infection for whom vaccination is highly recommended and administered free of charge. As for risk factors, beauty treatments including piercing and tattooing, unprotected sexual promiscuity, and being a family contact of an HBsAg carrier were among the strongest independent predictors of acute hepatitis B. Noteworthy, during the past decade (2010–2019), the number of IVDU patients with acute disease markedly decreased, while the number of those reported among foreigners significantly increased, currently accounting for nearly one-fifth of patients notified to SEIEVA [73].

In addition to the incidence, the prevalence of HBV markers of infection also decreased significantly after the introduction of vaccination. In this context, a population-based survey carried out in a highly endemic town of South Italy showed that the overall prevalence of HBsAg was 13.4% before vaccination and declined to less than 1%, 20 years after the implementation of vaccination; in addition, the prevalence of anti-HBc antibody (antibody to hepatitis B core antigen) dropped of about 10-fold (from 66.9% to 7.6%) in the same population and in the same interval of time between pre- and post-vaccination [74]. Additionally, the prevalence of anti-HBc antibody routinely detected in Italian recruits (20 years old) was 16% in those tested in 1981 and fell to less than 1% in those tested in 2020. Again, a serological study carried out between 2008 and 2009 in a large number (over 13,000) of pregnant women showed that the overall prevalence of HBsAg was 0.4%, but dropped to zero in women under 30 years of age (those covered by vaccination) [75]. In other words, thanks to vaccination, a large proportion of Italian people (about 25 million or 42 age cohorts) have almost no markers of ongoing HBV infection, because they are protected by immunization. Moreover, following vaccination, the number of HBV-related deaths declined by about 82%, from 9000 per year (or 15.5 per 100,000 people) in the late 1980s to around 1700 per year (or 2.84 per 100,000 people) in 2019 [76].

Finally, 30 years after the implementation of universal vaccination, in Italy, the pool of HBsAg carriers is numerically diminished by about three-quarters (declining from 1.5 to 2 million, or approximately 3% of the resident population in the pre-vaccination era, to the currently estimated 450,000 or about 0.7% of inhabitants) [77]. Noteworthy, most of these carriers are currently well over their fifth decade of life, with a long-lasting history of carrier state, and likely acquired infection several years ago (cohort effect) when they were younger and more prone to be infected with HBV.

## 6. Impact of Hepatitis B Vaccination on Delta Hepatitis

There is no specific vaccine against hepatitis Delta. Hepatitis Delta Virus (HDV) is a defective, single-stranded circular RNA virus, which requires the helper function of HBV to infect and replicate [78]. Given the biological association between these two viruses, HDV infection can occur either simultaneously with HBV (co-infection) or in chronic carriers of HBsAg (super-infection). Thus, an added benefit of vaccination is that protection against HBV can also be effective in protecting against hepatitis D when the two viruses are transmitted by co-infection. Indeed, according to SEIEVA data, in Italy the overall incidence of acute hepatitis delta per 1 million people declined from 3.2 cases in 1987 to 0.04 in 2019, in parallel to that of acute hepatitis B [79].

## 7. Conclusions and Future Prospects

Viral hepatitis type B is a major worldwide public health problem, being a leading cause of morbidity and mortality rates, as well as significant personal, societal, and economic costs. Following the WHO recommendations, 190 countries have successfully introduced universal infant vaccination programs on a global scale, and Italy was one of the first of these countries. Taken together, several billion vaccinations have been administered so far, with an outstanding record of safety and efficacy. Vaccines against hepatitis B are highly immunogenic and able to confer long-lasting immunity. Vaccine-induced anti-HBs antibody concentrations decline or even disappear over time, but immune memory to HBsAg persists beyond the loss of antibodies, providing long-term protection against acute and chronic disease [64,68,69,70,71,72,73]. Most vaccinees who lost humoral immunity usually show strong anamnestic responses when boosted 25–35 years after primary vaccination. No serious adverse events are associated with hepatitis B vaccination, and after administration of the routine three doses of vaccine, protection is achieved in over 95% of recipients. Benign breakthrough infections are occasionally detectable in successfully vaccinated individuals; the occurrence of acute clinical disease and the development of the carrier state are very rare events, but continued surveillance is necessary. Based on the current scientific evidence, the administration of boosters is not regarded as necessary to sustain long-term immunity in routine immunization programs involving healthy individuals [38,39,40,41,43,53,54,80,81].

Studies conducted in highly HBV endemic areas that were early to introduce and implement universal hepatitis B immunization (e.g., Alaska, Taiwan, and The Gambia) provided strong evidence of the hepatitis B vaccination effectiveness in reducing the incidence of disease, carrier rates, and HBV-related mortality, mainly due to cirrhosis and HCC [50,66].

Globally, remarkable progress in the introduction and implementation of vaccination has been achieved in recent years, but much work remains to meet the WHO targets of controlling hepatitis B in the community at large. To improve vaccination delivery, efforts are needed to undo social and economic barriers that still impede the administration of hepatitis B vaccination, especially in countries with low resources that are those at the highest HBV endemicity.

Vaccination has proved to be very successful in Italy. According to the SEIEVA data, the overall incidence rate of acute hepatitis B per 100,000 inhabitants dropped by over 96% in the past 30 years of universal vaccination. Moreover, seroprevalence studies show that a large proportion of our vaccinated population 42 years of age or younger have practically no markers of HBV infection (i.e., positivity for HBsAg and/or anti-HBc antibody). Finally, in addition to the remarkable decline in hepatitis B, hepatitis D also declined significantly, reflecting the impact of universal hepatitis B vaccination on both infections. This favorable trend towards the control and prevention of hepatitis B is destined to continue over time due, on one hand, to the universal vaccination that increases the immunized cohorts year after year, and on the other hand, to the fact that the current pool of HBV carriers is growing older and older, and thus at increased risk of numerically vanishing over time.

By January 2020, the WHO’s ETAGE (European Technical Advisory Group of Experts) of Immunization praised Italy for the significant results reached in the implementation of universal vaccination, which made the country one of the first in Europe to be validated for achieving the regional hepatitis B control targets [82]. To consolidate and improve these results, the working group recommends that Italy should continue conducting studies to evaluate coverage of hepatitis B screening for pregnant women and coverage of post-exposure prophylaxis for children born to infected mothers to ensure effective prevention of perinatal transmission of HBV on a national scale.

Italy’s priorities for the future include the maintenance of universal infant vaccination, the development of educational campaigns to increase the public’s understanding of prevention, to rationally convince people at increased risk of infection of the necessity of becoming vaccinated, and to facilitate the access of immigrants and refugees to health care services.

## Figures and Tables

**Table 1:** Hepatitis B vaccines.

Generation	Year	Type of Vaccine
I	1982	Plasma-derived vaccines
II	1986	Recombinant HBV DNA vaccine, expressed in yeast
III	1990	Recombinant pre-S/S vaccines expressed in mammalian cells
IV		Recombinant HBV vaccines with adjuvant (AS04, CpG)

## Data Availability

Not applicable.
